# Correction: Hernando-Ramiro et al. Time to First Fix Robustness of Global Navigation Satellite Systems: Comparison Study. *Sensors* 2025, *25*, 1599

**DOI:** 10.3390/s26175341

**Published:** 2026-08-24

**Authors:** Carlos Hernando-Ramiro, Óscar Gamallo-Palomares, Javier Junquera-Sánchez, José Antonio Gómez-Sánchez

**Affiliations:** Spanish National Institute for Aerospace Technology (INTA), Carretera de Ajalvir, km 4, 28850 Torrejón de Ardoz, Spain

## Error in Figure

In the original publication [1], there was a mistake in Figure 8 as published. Some of the values and, consequently, lines were wrong. The corrected Figure 8 appears below.

## Error in Table

There were mistakes in Table 3 as published. Some of the values in the table were wrong. The corrected Table 3 appears below.

## Text Correction

1. There was an error in Section 4.1, Paragraph 4. The content “On the other hand, its horizontal error is not significantly affected by attenuation until about 14 dB.” has been updated to “On the other hand, its horizontal error is not significantly affected by attenuation until about 12 dB.”

2. There was a typographical error in Section 4.2, Paragraph 3. The Content “However, regarding positioning accuracy, in those cases where a fix is achieved, the horizontal error obtaine is solid, providing values that are more precise than those of BeiDou, but larger than the GPS ones.” has been updated to “However, regarding positioning accuracy, in those cases where a fix is achieved, the horizontal error obtained is solid, providing values that are more precise than those of BeiDou, but larger than the GPS ones.”.

The authors state that the scientific conclusions are unaffected. This correction was approved by the Academic Editor. The original publication has also been updated.

## Figures and Tables

**Figure 8:**
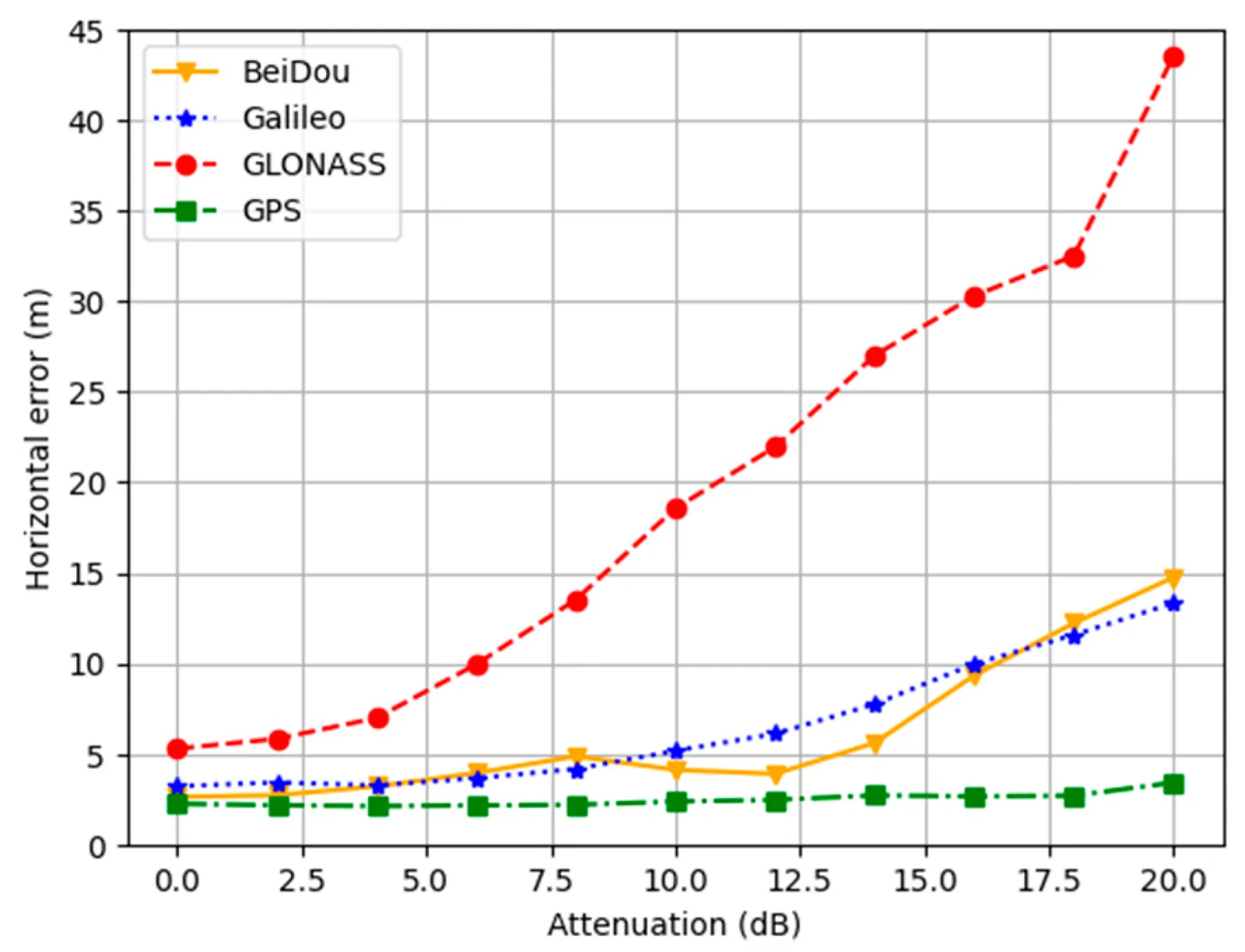
Horizontal error versus attenuation for the different GNSSs.

**Table 3:** Statistical results for the horizontal error.

Horizontal Error	GNSS	Attenuation (dB)										
		0	2	4	6	8	10	12	14	16	18	20
Mean (m)	BeiDou	2.62	2.72	3.20	3.94	4.86	4.12	3.89	5.61	9.34	12.2	14.8
	Galileo	3.21	3.43	3.26	3.66	4.17	5.16	6.12	7.74	9.97	11.6	13.3
	GLONASS	5.29	5.83	6.98	9.94	13.5	18.6	22.0	27.0	30.3	32.5	43.6
	GPS	2.24	2.16	2.11	2.15	2.19	2.37	2.46	2.71	2.66	2.69	3.41
Standard deviation (m)	BeiDou	1.62	1.71	2.96	5.18	7.51	3.23	2.60	5.38	17.9	19.4	20.0
	Galileo	3.65	4.12	3.79	4.65	5.33	7.10	8.23	9.39	11.3	11.5	11.7
	GLONASS	3.94	4.70	6.14	10.4	11.0	15.7	20.7	27.3	30.8	30.9	37.1
	GPS	1.47	1.37	1.32	1.35	1.36	1.60	1.78	1.79	1.71	1.83	2.30
Minimum (m)	BeiDou	0.22	0.37	0.44	0.50	0.42	0.67	0.70	0.54	0.74	0.94	2.03
	Galileo	0.25	0.32	0.46	0.63	0.62	0.71	0.64	0.52	1.22	1.52	1.52
	GLONASS	0.62	0.71	1.10	1.56	1.68	1.78	4.28	4.04	2.59	3.69	3.49
	GPS	0.21	0.21	0.22	0.26	0.41	0.23	0.40	0.85	0.59	0.58	0.99
Maximum (m)	BeiDou	11.5	10.7	26.3	48.6	66.4	21.2	13.4	32.3	163	163	163
	Galileo	29.3	31.0	26.5	27.4	36.6	43.1	43.1	54.6	59.2	59.2	59.2
	GLONASS	30.5	37.5	46.8	63.3	69.2	72.2	114	155	173	155	226
	GPS	8.30	6.71	6.45	7.26	7.59	8.45	10.5	10.3	9.80	12.6	17.0
